# Gender-differences in conservatoire music practice maladjustment. Can contextual professional goals and context-derived psychological needs satisfaction account for amotivation variations?

**DOI:** 10.1371/journal.pone.0232711

**Published:** 2020-05-04

**Authors:** Rafael Valenzuela, Nuria Codina, José Vicente Pestana

**Affiliations:** Department of Social Psychology and Quantitative Psychology, University of Barcelona, Barcelona, Spain; University of Macau, MACAO

## Abstract

In music education, women are present in great numbers. In professional settings, however, women musicians are not as predominant. With some exceptions, such as Scandinavian countries, women still pursue gender equality in professional music practice. To inquire about the causes of this, we considered if gender-differences in amotivation in conservatoire instrument practice could be associated with aspects of learning environment. Self-determination theory (SDT) posits that learning environments may influence motivation, by satisfying or thwarting students’ psychological needs and by selectively endorsing specific extrinsic goals. Thus, we analysed if–women and men–amotivation variations could be explained by differences in behavioural regulations and satisfaction of their psychological needs for competence and autonomy. Participants (67 women and 74 men, 18–47 years old) completed validated scales for amotivation, behavioural regulations, and needs satisfaction. Students exhibited high intrinsic and introjected regulations, and high autonomy and competence needs satisfaction. Students’ identified regulation levels were modest, and external regulation and amotivation levels were low. Women students’ perceived competence was lower, and their amotivation was higher than men’s. Amotivation variations were explained positively by identified regulation and negatively by context-derived satisfaction of the psychological needs for competence (and autonomy, only among women). Results suggest that internalization of extrinsic goals can pose difficulties and that psychological needs satisfaction may counteract amotivation (autonomy being potentially more important for women musicians).

## 1. Introduction

Conservatoire musicians tend to initiate their music education early in life, given that reaching the indispensable performance levels requires perseverance to practise regularly and frequently for a long time [1]. In order to achieve this kind of engagement in music education, typically, their instrument practice is deeply intertwined with personal identity, sense of self and intrinsic motivation [2]. In this regard, self-determination theory (SDT), an organismic theory of human motivation, behaviour and personality development [3], has an important say. Meanwhile, in music education, women are at present in great numbers, but in professional settings, women musicians are not as predominant. With some exceptions, such as Scandinavian countries, women still pursue gender equality in professional music practice. To inquire about the causes of this, we considered if gender-differences in their motivation in conservatoire instrument practice could be associated with aspects of learning environment from the perspective of SDT, specifically, in amotivation–a lack of intention to act.

Evans [4] presented a conceptual review of a self-determination theory approach to motivation in music education, wherein he has summarized several advantages of adopting an SDT perspective for music education. First, SDT provides considerable breadth which enables us to explain a wide range of behaviour and factors of interest in studying motivation for music learning, such as self-efficacy [5], resilience [6], achievement goals [7] and so forth. Further, persistence and resilience has been an important subject of research in music education. They have been examined using SDT in sports [8], mental health [9] and school contexts [10]. Third, SDT goes beyond the quantity of motivation, yet places a strong emphasis on the quality of motivation and behaviour. This is particularly important for developing musicians, for example, in the extent to which practice needs to be deliberate and effortful [11], and of good quality [12]. These advantages altogether lay a strong theoretical ground for the current study, which sought to examine the different behavioural regulations in conservatoire music practice.

## 2. Literature review

SDT is an organismic theory of human motivation, behaviour and personality development, which posits that people have a natural tendency towards psychological growth, integration, and well-being. The theory argues that behaviour is regulated by different types of motivation (behavioural regulations), which exist on a continuum from controlled to autonomous, the latter being the best quality of motivation, given its high degree of alignment with the self. Following SDT, learning environments and social-contextual factors can promote (or hinder) people’s quality motivation, through satisfying (or thwarting) their psychological needs for competence, autonomy and relatedness. Given that, in terms of SDT, music students’ motivation can be internalized (this is, aligned with the self) only to the extent that their psychological needs are fulfilled, it is necessary to investigate motivation in higher music education taking into account the complex interplay between learning environment, psychological needs satisfaction (PNS), and the quality of the resulting behavioural regulations.

### 2.1. Psychological needs satisfaction and behavioural regulations in the music conservatoire

Conservatoire musicians tend to initiate their music education early in life, given that reaching the indispensable performance levels requires perseverance to practise regularly and frequently for a long time [1]. In order to achieve this kind of engagement in music education, typically, their instrument practice is deeply intertwined with personal identity, sense of self and intrinsic motivation [2].

In this regard, self-determination theory (SDT), an organismic theory of human motivation, behaviour and personality development [3], has an important say, given that, in addition to being rooted in the construct of ‘intrinsic motivation’, this theory argues that people have a natural tendency towards psychological growth, integration, and well-being [13], which can only flourish if their universal psychological needs for competence, autonomy and relatedness get satisfied by the environments in which they act. According to SDT, behavioural regulations (and also dysregulations, like amotivation) result from the interplay between environments with their unique characteristics and persons with their unique interests and skills.

If the environment of the activity facilitates psychological needs satisfaction (PNS), people may stand behind their behaviours to a greater extent and produce quality motivation [14]. Contrarily, if environments thwart these needs, motivation recedes and becomes sub-par. Social environments, such as schools and conservatoires, can promote quality motivation, by facilitating satisfaction of these needs, which are considered universal psychological nutriments that allow people to strive and produce their best performance and well-being [15]. Conservatoire students, for instance, may perceive their psychological needs to be satisfied when [1]: they are able to choose how to practise their instrument or to voice their opinion (autonomy need); they manage to fulfil the goals of one lesson or master one piece (competence need); and when they feel closely related to their classmates or teachers (relatedness need).

In the in the present work, we focus on the satisfaction of the needs for autonomy and competence, given that these two needs have been reported to be sensitive to day-to-day within-person fluctuations [16]. Conservatoire students’ levels of perceived competence and autonomy in instrument practice are assumed to be robust, given that perceived autonomy plays a critical role in vocational pursuits strongly based on intrinsic motivation [2] and that intrinsic motivation is deeply rooted in perceived competence [17]. We, thus, put the focus on the satisfaction of these two task-relevant needs, based on the notion that, as opposed to other conceptualizations, needs in SDT are not organized into a hierarchy and all three contribute unique proportions of explanation in favourable outcomes such as motivation [16].

Specific links between PNS and quality motivation are observable. Insofar a person endorses an activity as interesting or enjoyable their need for autonomy is satisfied, thus, enabling autonomous motivation [16]; on the contrary, autonomy need frustration leads to alienation and diminishes intrinsic motivation. Furthermore, because people rarely enjoy activities that make them feel incompetent, they practise intrinsically motivating activities, not only out of interest, but also to feel competent at doing so [17]. Consequently, environments can foster quality motivation, by facilitating perceived competence and autonomy [1, 2, 4, 18,19]; contrarily, adverse social-contextual aspects, like poor teaching style, may thwart psychological needs and lead to amotivation [20].

To be more exacting about the different ways in which behaviour is regulated, according to SDT, intrinsic motivation (IM) is defined as the drive to participate in an activity, motivated by the enjoyment of the participation itself (rather than by its extrinsic outcomes); whereas, contrastingly, extrinsic motivation (EM) refers to behavioural regulation derived from the pursuit of goals extrinsic to the activity itself, but which result from it [14]. Nevertheless, extrinsic motives can also be autonomously endorsed, given that people often willingly perform uninteresting behaviours, as long as these are instrumental in reaching extrinsic goals of high personal importance [17]. This kind of extrinsic-yet-autonomous regulation is called identified regulation (ID), because people feel identified with those goals, even if activities entailed in their accomplishment are unattractive: for instance, a conservatoire student may practise scales, not because they enjoy it *per se*, but because they highly value strengthening their advanced skills. Thus, identified regulation is considered high-quality motivation, because it enables people to regulate their behaviour towards future-oriented extrinsic-yet-autonomous goals of high importance to the self (even if these lead to activities unattractive in the present) [21].

These two regulations, identified regulation (i.e. practising for extrinsic–yet autonomous–personal goals), alongside intrinsic motivation (i.e. practising for the interest, curiosity or enjoyment of practice itself), integrate a higher order composite called autonomous motivation [14], of which intrinsic motivation is the most typical form [17, 22]. Autonomous motivation has been considered of great importance for young musicians [23] and it has been linked with positive outcomes like flow in conservatoire music practice [24]. Furthermore, when people regulate their behaviour autonomously, they tend to perform better and experience wellbeing [25]. This means that they autonomously endorse said behaviours at the highest order of reflection, not just that they use their personal control to perform these behaviours demanded by others without understanding the rationales behind these actions. Consequently, SDT argues that autonomous motivation (IM + ID) is of better quality than controlled motivation. The latter consists of introjected regulation (IN) (i.e. behaviours regulated by introjected heteronomous standards, performed with internal control but, generally, to avoid feelings of guilt or shame) and external regulation (ER) (i.e. behaviours regulated by approaching or avoiding, respectively, favourable or adverse external consequences, such as gains or detriments). Both of these regulations have low levels of autonomy and, thus, may promote conformity, thwart self-expression, and result in feelings of alienation and motivation of lesser quality [16]. Nevertheless, as people progress through their careers in music, the proportions of diverse intrinsic and extrinsic motives may vary: for instance, motives like expressing a musical identity, satisfying personal needs, approaching learning effectively, or addressing specific environmental conditions may, respectively, gain more salience during specific periods, consequently, affecting motivation and possibly even individual career trajectories [26]. Students’ experiences may vary not only due to the specific moment in their music career, but also students with different motivational goals may experience their learning environments differently, because they direct their attention to different aspects [27]. As a result, under certain circumstances, the presence of extrinsic motives has been reported to pose challenges to the continuity of intrinsic or autonomous motivation, by veering attention towards externally mediated gains or rewards [22, 28]. For instance, highly performance-centred environments, foster high competence levels, through hetero-normative standards, based on criteria external to the person. Furthermore, emphasis on performance, grade evaluations, extrinsic motives and external control may disrupt students’ natural tendency towards learning [29], replacing enthusiasm and interest by boredom, anxiety and alienation [25], thus, negatively affecting people’s autonomous motivation by thwarting their senses of agency and ownership over their behaviour [30].

This coexistence of diverse motives in higher music education presents a challenge for music educators. Regarding the positive or adverse outcomes of combining intrinsic and extrinsic motivational goals, meta-analytic research [31] has specified conditions under which people tend to interpret their intrinsic and extrinsic motives as working together, or against each other: both kinds of motives seemed to oppose each other when intrinsic motivation was assessed as a free-time measure (over-justification hypothesis); whereas they seemed to work additively to produce quality motivation (additive hypothesis) when intrinsic motivation was evaluated as a performance-based measure [31]. In other words, extrinsic motives could predict lesser free engagement in activities, but not necessarily lesser enjoyment of performance-based activities that are being practised anyway. Thus, notwithstanding the caveats of a potential opposition between intrinsic and extrinsic motivation [28], it has also been argued that, under some circumstances, they can behave additively and produce high quality motivation [31]. For example, controlled regulations have been reported to coexist with intrinsic motives in long-time vocational engagement where performance stands out as very important; and even external motives have been reported to coexist with internalized motives, producing quality motivation, insofar internalized motives (and not only external ones) are high [23].

In music education, seemingly, the problem arises, not when controlled regulations are present [23], but when psychological needs are unfulfilled, since this shortfall has been linked with dropout [20]. However, conservatoire teachers tend to be not only demanding of externally-referenced performance standards, but also controlling [32] and prescriptive of the ways to accomplish them; thus, leaving students with limited opportunity to feel that they can influence the ways in which they are taught [33]. Contrastingly, it is PNS (including autonomy need satisfaction) and autonomous motivation which have been related with lower negative and higher positive emotions, with higher preference for challenges, with higher frequency of both practice and practice of high quality [34], and lastly with intentions to continue practising; hence, PNS should be taken into consideration when facing problems of retention and dropout [1].

Furthermore, PNS can also play important roles in promoting additive relationships between diverse motives. For instance, *internalization* of extrinsic motives (the process by which behaviours that at first may be subject to external regulation, may then become aligned with the self), cannot happen without the indispensable autonomy need satisfaction to allow for this self-alignment [4]. In other words, if students have previously incorporated goal beliefs that are aligned with those of their teachers or learning environments, then their own goals may *identify* with context demands more easily, without a sense of loss of autonomy [16]. This further attests to the importance of explaining rationales to the students and of paying attention to aspects of learning environments that could set specific conditions for PNS and its influence on quality motivation.

When learning environments or social-contextual factors thwart people’s psychological needs, then quality motivation is hindered [14, 35] and, consequently, dysregulations like amotivation become more probable. From the perspectives of both researchers and practitioners, however, understanding and assessing amotivation are complex tasks. It has been defined as a lack of intention to act, derived from not valuing the activity, or from feeling incompetent at it, or from having low expectations of the results [14]. Several studies have focused on amotivation in diverse activity domains. In high school academic learning, it has been described as a form of behavioural dysregulation, characterized by a dissociation between intentional behaviour and expected outcome [36]. And in physical education, as a lack of perceived connection between finalistic action and intended results [37]. When people experience *amotivation* they feel disintegrated from their activity, cease to invest energy in it, and may desist of it completely [14]. Lastly, amotivation measures have been linked with adverse contextual factors, like poor teaching style [38], unattractive or unvalued tasks, and adverse personal ability or effort beliefs [37]. These findings highlight that amotivation is a multifaceted construct, in which both personal and contextual factors may be relevant.

### 2.2. Gender-differences in conservatoire music practice

Gender-differences have been studied in diverse fields, including personality, with early work reporting that women scored higher than men in anxiety, extraversion, and nurturance, but not in locus of control, orderliness, reflexivity, and impulsiveness [39]. More recent studies have shown that gender-differences in explicit personality (self-reports) seem to be larger than in implicit personality (based on implicit associations), and that women’s implicit agreeableness and neuroticism are higher than their male’s counterparts, whereas men score higher on implicit extraversion and openness [40]. As regards attitudes towards learning, task value ascribed to diverse subjects has been reported to manifest gender-differences, with women valuing languages, arts, and biology higher and physics lower than their men counterparts [41]. Furthermore, also impulsivity has been studied from the perspective of gender-differences, with men students reporting higher positive urgency and sensation seeking and marginally lower perseverance [42].

As regards cognitive styles, in western societies, men have been found to favour systemizing styles (i.e. focus on rule-based systems and their prediction), whereas women have been found to favour empathizing styles (i.e. focus on the mental states of others, behaviour prediction and the generation of appropriate emotional responses), producing gender disparities in various domains [43]. Furthermore systemizing style is more prevalent in the sciences, whereas empathizing style in the humanities, thus potentially attracting like-styled individuals [43]. Women students, however, have been reported to feel less efficacious overall [44] and to conform more to teachers’ standards and norms [45]. Women students have also been reported to make higher stress appraisals, which may lead them to maladaptive disengagement coping strategies, which are closely related with career intentions [46].

In advanced music practice, women seem to be more prone to negative experiences, such as anxiety and performance anxiety [47]. Thus, maintaining high levels of motivation, indispensable for conservatoire studies [48], may be more difficult for some women musicians. Women and men may experience conservatoire studies differently, given that gender-differences in music education are manifold and complex, spanning across diverse aspects such as instrument choice [48], learning experiences [49], and even listening preferences [50]. Also, motivations for studying music may be related with gender, as women ascribe more task difficulty, usefulness and importance to music practice [51].

Furthermore, gender may exert some influence on learning processes, given that men and women musicians approach higher music education differently. Men musicians tend to attribute higher importance to drive for musical excellence, to use more critical thinking strategies while evaluating their performance [52], and to consider analytical musical skills more representative of musical expertise than women [53]. They also do warm-up exercises more often and keep concentration more frequently, without stopping to correct mistakes immediately [54]. Lastly, when they prepare for performance, their practice is less structured and more pragmatic [55].

Alternatively, women students use self-regulated learning strategies more often and ascribe more importance to musical skills [55]. They, autonomously take more structured, rigorous, strict or self-demanding pathways in music practice and are more concerned with coping skills, however, scoring lower on these than men [53]. Women musicians conform more to teachers’ standards and norms [45] and use systematic practice strategies more often, such as repeatedly playing passages slowly, setting targets, making practice lists, starting their practice with scales, marking difficult passages on the parts, and stopping to correct mistakes immediately [54]. However, sometimes they may feel less efficacious overall [44] and it has been suggested that they may experience a larger gap between their ideal and self-perceived skill levels, thus, having less positive attitudes towards their own skills [55]. Women music students also tend to make higher stress appraisals, which–added to the adverse influences of the typically controlling teaching style in conservatoires–may lead them to maladaptive disengagement coping strategies, which are closely related with career intentions [46].

Gender, in general, can shape various psychological aspects implied in education, such as attitudes towards learning [55] and cognitive style [43], producing gender disparities in various domains. And lastly, some voices have also argued that biased constructions about gender and music practices–present in higher music education institutions–may be discouraging for some women musicians [56, 57]. The aspects mentioned above may be leading women musicians to a greater risk of amotivation and dropout, through negative experiences like stress [46] and performance anxiety [53].

### 2.3. The present study

Based on the aforementioned antecedents, it is relevant to investigate the relationships between different behavioural regulations and context-derived PNS in conservatoire music practice, and to analyse their conjoint effects on amotivation variations. Similar examples exist in sports practice, analysing the conjoint effects of achievement motives and competence need satisfaction on subsequent intrinsic motivation [19], and also in school settings, describing the conjoint effects of intrinsic motives, performance motives, and perceived performance on well-being [58]. Furthermore, the concern that women musicians may be more prone to amotivation in the music conservatoire has come from studies which have focused mainly on gender-differences in individual variables, such as learning strategies and self-perceived skill levels [45, 52, 53, 55]. Less common, however, have been studies considering if contextual influences (such as PNS, derived from learning environments, and extrinsic performance motives, implied in professional goals) may affect women and men motivation quality in diverse ways, setting women musicians at higher risk of dysregulation and dropout.

Taking into consideration all the arguments mentioned above, we asked if amotivation variations in conservatoire music practice could be explained conjointly by four behavioural regulations (intrinsic, identified, introjected, and external) and by PNS of the needs for competence and autonomy. Furthermore, we considered if such an explanation would be subject to gender-differences. As regards the hypotheses of the present study, we anticipated intrinsic motivation to yield high scores (H_1_) [2] and amotivation low scores (H_2_). Nonetheless, we expected amotivation to be more pronounced among women (H_3_) [53]. Furthermore, we expected intrinsic motivation to be positively correlated with perceived competence (H_4_) [8, 9] and amotivation variations to be negatively explained by perceived competence (H_5_) and autonomy (H_6_) [13–15, 35, 59]. Also, given their higher tendency towards autonomous self-regulation, we expected that women students’ autonomy need satisfaction (as compared to men’s) would play a more pronounced role in explaining amotivation variations (H_7_) [44, 46, 53]. Lastly, we expected that extrinsic motives, gaining salience in professional education, such as identified regulation towards professional goals and even controlled regulations would be also present alongside robust levels of intrinsic motivation in instrument practice [23].

Furthermore, as a mean of controlling for confounding effects of type of instrument played, we registered this variable and analysed gender-differences within different instrument practices that included sufficient participants for comparison. This control seems reasonable, given that it has been argued that instrument of choice may play a role in determining teaching style, which may in turn influence perceived autonomy [59]: for example, guitar students tend to report higher autonomy and willingness to play than piano students; and a more rigid teaching style was observed in piano than in guitar studies.

## 3. Materials and methods

### 3.1. Participants

Participants were 141 conservatoire students enrolled in mandatory instrument practice, of whom 74 identified as men and 67 as women. Their ages (*M* = 22.5, *SD* = 4.4) ranged from 18 to 47, though the majority was 24 years old or younger (80%). Seven were not included due to not reporting sex and four due to not reporting age. As the sole public conservatoire of the city, admission rates (per instrument) are limited (compared to demand) and in a typical case, an 18-year-old student facing entrance examinations started their music education around the age of six.

Two thirds of participants (*n* = 94; 66.7%) studied to become instrument performers, whereas *n* = 22 (15.6%) studied music pedagogy, *n* = 8 (5.7%) musicology, *n* = 8 (5.7%) sonology, *n* = 6 (4.3%) composition, and *n* = 3 (2.1%) production. As regards type of musical background, *n* = 97 (68.8%) studied classical, *n* = 23 (16.3%) jazz and modern, *n* = 9 (6.4%) antique, and *n* = 12 (8.5%) did not report musical background. Furthermore, regarding instruments played the most, *n* = 34 participants (24.1%) played the piano, *n* = 16 (11.3%) violin, *n* = 14 (9.9%) guitar, and *n* = 9 (6.4%) C-flute. Participants studying vocals and cello were, respectively, *n* = 7 (5.0%); whereas participants playing clarinet, horn, and trumpet were, respectively, *n* = 6 (4.3%). Other instruments played were contrabass (*n* = 5); saxophone (*n* = 4); drums, fagot, and viola (respectively, *n* = 3); harp, electric bass, oboe, percussion, and trombone (respectively, *n* = 2); and accordion and other traditional or antique instruments (respectively, *n* = 1).

### 3.2. Procedure

The conservatoire’s head of research granted institutional approval for the study. Sampling method was intentional and all participants had to be attending mandatory instrument lessons (vocals counted as instrument). Students were contacted through their teachers (who participated voluntarily) and were delivered the instruments as one last extra section of their regular course evaluations that they could complete voluntarily and anonymously. Participants received instructions in writing and read out loud by their teachers, including informed consent regarding the purpose of the study, confidentiality, absence of negative consequences of declining to participate, their right to withdraw at any time, and contact information of the researchers.

The ethical requirements of the Ethics Committee of the University of Barcelona (University of Barcelona's Bioethics Commission, CBUB–Institutional Review Board IRB00003099) were applied to the current study, which meant that additional approval for the research was not required because the data obtained did not involve animal or clinical experimentation. Additionally, this study complies with the recommendations of the General Council of Spanish Psychological Associations (Consejo General de Colegios de Psicólogos), the Spanish Organic Law on Data Protection [60] and the Declaration of Helsinki [61].

### 3.3. Measures

#### 3.3.1. Behavioural regulations and amotivation

Four types of behavioural regulations were assessed with the Spanish translation [62] of the Sport Motivation Scale (SMS) [63]. This measure is derived from a previous French-Canadian measure–*L'Échelle de Motivation dans les Sports (ÉMS)* (Pelletier, Fortier, Vallerand, Tuson, Brière, & Blais, 1995) [64]–based on concepts derived from self-determination theory [13–16, 35, 59]. It includes seven subscales, comprising four items each, respectively, measuring intrinsic motivation to experience stimulation, intrinsic motivation to know, intrinsic motivation towards accomplishment, identified, introjected and external regulations, and also amotivation. The stem was rephrased as “why do you practise your instrument?” instead of the original “why do you practise your sport?”. Sample items for each subscale include the following: “for the pleasure (enjoyment) I feel in living exciting experiences” (IM-stimulation), “for the pleasure (enjoyment) it gives me to know more about the instrument that I practise” (IM-to know), “because I feel a lot of personal satisfaction while mastering certain difficult practice techniques” (IM-accomplishment), “because it is one of the best ways I have chosen to develop other aspects of myself” (identified regulation), “because I would feel bad (about myself) if I was not taking time to do it” (introjected regulation), “for the prestige of being a musician” (external regulation), and “I used to have good reasons for practising my instrument, but now I am asking myself if I should continue doing it” (amotivation).

Participants rated each item using a seven-point Likert scale, ranging from “not at all like me” (1 point) to “totally like me” (7 points). Research with Spanish respondents has previously confirmed reliability of the seven subscales, with Cronbach’s alphas between .74 and .83 for five out of the seven subscales, and alphas of .68 and .64, respectively, for identified and introjected regulations [65]. Cronbach’s alphas in this study were acceptable for each subscale (ɑ_IM-stimulation_ = .83; ɑ_IM-know_ = .80; ɑ_IM-accomplishment_ = .81; ɑ_EM-identified_ = .70; ɑ_EM-introjected_ = .85; ɑ_EM-external_ = .83; ɑ_Amotivation_ = .77).

#### 3.3.2. Psychological needs satisfaction of the needs for autonomy and competence

To assess PNS students were asked about their “experience in instrument lessons and its derived practice”. Autonomy need satisfaction was assessed using the Spanish translation [62]; of the 10-item perceived autonomy scale (Cronbach’s alpha of .89) [63], the anchor was “In my instrument lessons”, and it included items like “I feel that my choices and actions are based on my true interests and values”. Cronbach’s alpha in this study was acceptable (ɑ = .91). Competence need satisfaction was measured with the Spanish version [62] of the five-item perceived competence subscale, derived from the Intrinsic Motivation Questionnaire [66]. The anchor was “While I am practising with my instrument”. Items such as “I think I am pretty good at my sport” were rephrased as “I think I am pretty good at playing my instrument”. In validation studies with Spanish respondents this subscale yielded Cronbach’s alphas of .80 [66] and of .79 [62]. Cronbach’s alpha in the present study was acceptable (ɑ = .82).

### 3.4. Analyses

Statistical analyses were performed with IBM SPSS Statistics for Windows (Version 24.0) and RCommander (R package). All study variables were described including means, standard deviations and gender-differences (Table 1). Also, bivariate correlations between variables were reported (Table 2). Finally, multiple regression analyses were performed to assess the proportion of amotivation variations explained conjointly by behavioural regulations and psychological needs satisfaction among men (Table 3) and women (Table 4).

**Table 1:** Means, standard deviations and gender-differences in conservatoire students’ behavioural regulations, amotivation, and psychological needs satisfaction (autonomy and competence).

	All		Women		Men			
	(*N* = 141)		(*n* = 67)		(*n* = 74)			
Variable	*M*	*SD*	*M*	*SD*	*M*	*SD*	*t*	*P*
Amotivation	2.28	1.20	2.74	1.42	1.86	0.74	-4.53	< .001
IM experience stimulation	5.30	1.19	5.33	1.10	5.28	1.28	-0.27	.790
IM to know	5.13	1.11	4.98	1.14	5.26	1.08	1.49	.138
IM accomplishment	5.01	1.22	4.94	1.23	5.07	1.23	0.66	.510
Identified regulation	3.78	1.17	3.79	1.06	3.77	1.27	-0.09	.927
Introjected regulation	4.89	1.40	4.88	1.37	4.89	1.43	0.03	.978
External regulation	2.88	1.43	2.81	1.39	2.93	1.47	0.61	.545
Perceived autonomy	4.51	0.81	4.39	0.88	4.62	0.72	1.67	.098
Perceived competence	4.27	0.81	4.04	0.85	4.48	0.71	3.40	.001

IM: intrinsic motivation. Possible scores for behavioural regulations: 1 through 7 points; and for psychological needs satisfaction: 1 through 6 points.

**Table 2:** Correlations between conservatoire students’ amotivation, behavioural regulations, and psychological needs satisfaction (competence and autonomy).

Variables	1	2	3	4	5	6	7	8
Amotivation								
IM experience stimulation	-.02							
IM to know	-.04	.62**						
IM accomplishment	-.05	.66**	.78**					
Identified regulation	.26**	.43**	.45**	.47**				
Introjected regulation	-.02	.39**	.59**	.62**	.36**			
External regulation	.05	.18*	.32**	.40**	.44**	.46**		
Perceived autonomy	-.33**	.06	.17*	.11	.01	.08	-.01	
Perceived competence	-.52**	.31**	.22**	.30**	.04	.17*	.21*	.29**

*N* = 141. IM: intrinsic motivation.

* *p* < .05 (two-tailed)

** *p* < .01 (two-tailed).

**Table 3:** Regression analysis for men students' amotivation variations, predicted by behavioural regulations and psychological needs satisfaction.

Model and predictor variable	Men					
	*B*	*SE B*	95% CI	β	*t*	*P*
Identified regulation	0.21	0.06	[0.08, 0.34]	.36	3.32	.001
Perceived competence	-0.26	0.11	[-0.49, -0.04]	-.25	-2.30	.024

CI = confidence interval for *B*. Models based on backward deletion method.

**Table 4:** Regression analysis for women students’ amotivation variations, predicted by behavioural regulations and psychological needs satisfaction.

Model and predictor variable	Women					
	*B*	*SE B*	95% CI	β	*t*	*p*
Perceived competence	-0.85	0.17	[-1.18, -0.52]	-.51	-5.15	.001
Identified regulation	0.38	0.12	[0.13, 0.63]	.28	3.08	.003
Perceived autonomy	-0.39	0.16	[-0.71, -0.07]	-.24	-2.45	.017

CI = confidence interval for *B*. Models based on backward method.

## 4. Results

### 4.1. Descriptives

Table 1 shows means, standard deviations, and gender-differences in conservatoire students’ amotivation, behavioural regulations, and psychological needs satisfaction of the needs for autonomy and competence in music practice. As regards behavioural regulations (Table 1), reports of intrinsic motivation were high (H_1_ substantiated), as expected in a vocational setting as the music conservatoire. Introjected regulation, however, also received surprisingly high reports. Identified regulation yielded mean scores close to neutral, and external regulation mean scores were low. As anticipated, amotivation was low (H_2_ substantiated), but women students’ amotivation levels (*M* = 2.74, *SD* = 1.42) were more variable and significantly greater (*t* = -4.53, *p* < .001) than men’s (*M* = 1.86, *SD* = 0.74) (H_3_ substantiated). As regards psychological needs satisfaction, perceived competence and autonomy were high, however, men students’ perceived competence (*M* = 4.48, *SD* = 0.71) was marginally higher (*t* = 3.40, *p* = .001) than their women counterparts’ (*M* = 4.04, *SD* = 0.85).

In order to screen for potential relations between gender, instrument played and study variables, we assessed gender-differences within studies with the highest counts (that allowed for comparisons). Among piano students (17 women and 17 men), women (*M* = 2.71, *SD* = 1.35) reported greater amotivation (*t* = -2.643, *p* = .014) than men (*M* = 1.78, *SD* = 0.73), however, amotivation among women was highly variable as observed in a high skewness. Women also showed lower (*t* = 2.315, *p* = .027) perceived autonomy (*M*_women_ = 3.99, *SD* = 0.89; *M*_men_ = 4.60, *SD* = 0.61); and marginally lower (*t* = 1.766, *p* = .087) perceived competence (*M*_women_ = 3.97, *SD* = 0.78; *M*_men_ = 4.47, *SD* = 0.86). Among students of violin (11 women and 5 men) and C-flute (5 women and 4 men) no gender-differences were observed in study variables. Among the 14 guitar students only two were women and they scored higher on amotivation (*M*_women_ = 3.97, *SD* = 0.78; *M*_men_ = 4.47, *SD* = 0.86) and lower than men in all behavioural regulations and psychological needs satisfaction, though the only gender-difference that reached significance levels was the one regarding women’s lower (*t* = 4.608, *p* = .001) intrinsic motivation to know (*M*_women_ = 3.88, *SD* = 0.18; *M*_men_ = 5.48, *SD* = 1.13).

### 4.2. Associations between study variables

Bivariate Pearson correlations (Table 2) revealed strong internal consistency between dimensions of intrinsic motivation. Interestingly, aligned with SDT’s predictions, the magnitude of the correlation coefficients between intrinsic motivation to experience stimulation (the aspect of IM most proximal to pure enjoyment) and extrinsic regulations followed a gradient, where identified regulation (the aspect of extrinsic motivation most proximal to the self) showed the highest positive association with IM; whereas introjected and external regulations, respectively, yielded consecutively smaller associations. Inversely, external regulation (the least autonomous regulation), was associated robustly with introjected regulation (the other controlled regulation); whereas identified and intrinsic regulations (the most autonomous regulations), respectively, yielded consecutively smaller correlations with external regulation.

Psychological needs satisfaction of the needs for autonomy and competence showed moderate internal consistency and were correlated with behavioural regulations (Table 2). Perceived autonomy was only associated positively with intrinsic motivation to know; however, as anticipated, perceived competence was positively associated with all three aspects of intrinsic motivation (H_4_ substantiated). Interestingly, perceived competence was also (positively) related with extrinsic motives, such as introjected and external regulations. For its part, as expected, amotivation was negatively associated with the satisfaction of psychological needs for competence (H_5_ substantiated) and autonomy (H_6_ substantiated, only among women). Surprisingly, however, identified regulation was positively associated with amotivation and was not associated with PNS.

### 4.3. Explanation of amotivation variations

The proportion of inter-subject amotivation variations explained conjointly by behavioural regulations (i.e. intrinsic, identified, introjected, and external) and by psychological needs satisfaction (i.e. perceived competence and autonomy) were assessed with multiple linear regression analyses. We used backward deletion method, because it first computes all effects simultaneously and subtracts the smallest insignificant effects one by one until there are only significant predictors left [67]. In order to explain amotivation variations, controlling for respondents’ sex and age, firstly, we ran multiple regression analyses on amotivation, including these variables as predictors. Age was statistically insignificant and, thus, was excluded from further analyses. Sex, nonetheless, was a significant predictor of amotivation (β = .57, *t*(137) = 3.50, *p* = .001), and amotivation was greater among women than among men (Table 1), which led us to describe two gender-differentiated models.

#### 4.3.1. Explanation of men students’ inter-subject amotivation variations

As regards tests of statistical assumptions, among men students, tolerance tests and variance inflation factor (VIF) of predictor variables showed acceptable levels to confirm that no collinearity problems occurred for identified regulation (Tolerance = .99, VIF = 1.01), and perceived competence (Tolerance = .99, VIF = 1.01), given that none of the tolerance coefficients was below questionable levels of tolerance (< 0.2) [68] nor any of the mean variance inflation coefficients was higher than 1.5 [69]. Heteroscedasticity was discarded given that Breusch-Pagan test (BP) indicated homoscedasticity along the criterion variable among men students (BP = 1.4412, *df* = 1, *p* = 0.23). As a result, given VIF coefficients indicating no multicollinearity, the model can serve for explanatory and predictive purposes, allowing for the estimation of an amotivation coefficient for men, based on the coefficients of predictor variables. Among men (Table 3), the proportion of amotivation variations explained was a modest *R*^2^ = .17, *R*^2^_adj_ = .15, *S* = 0.68 (*F*(2, 72) = 7.37, *p* < .01), and the model included identified regulation and perceived competence (H_5_ substantiated) as significant predictors of amotivation variations.

#### 4.3.2. Explanation of women students’ inter-subject amotivation variations

As regards tests of statistical assumptions, among women students, tolerance tests and variance inflation factor (VIF) of predictor variables also showed acceptable levels, confirming that no collinearity occurred for perceived competence (Tolerance = .85, VIF = 1.17), identified regulation (Tolerance = .98, VIF = 1,02), and perceived autonomy (Tolerance = .84; VIF = 1.19). Furthermore, Breusch-Pagan test (BP) indicated homoscedasticity along the criterion variable (BP = 1.8943, *df* = 1, *p* = 0.168). As a result, given VIF coefficients indicating no multicollinearity, the model can serve for explanatory and predictive purposes, making it possible to estimate an amotivation coefficient for women based on the coefficients of predictor variables.

Among women (Table 4), the proportion of amotivation variations explained was a robust *R*^2^ = .48, *R*^2^_adj_ = .46, *S* = 1.05 (*F*(3, 64) = 19.46; *p* < .001), and the model included three significant predictors of amotivation variations. As in the case of men, identified regulation predicted amotivation variations positively and perceived competence (H_5_ substantiated) predicted these variations negatively. In the case of women musicians, also perceived autonomy need satisfaction explained amotivation variations negatively (H_6_ substantiated), however this effect size was smaller than the effect size of the other two predictors, signalling that thwarted autonomy need could be explaining at least part of amotivation variations in women musicians over and above identified regulation and perceived competence. Perceived autonomy, which was more variable among women than men (see Table 1), explained a greater proportion of amotivation variations among women than among men, as hypothesized (H_7_ substantiated); in fact, it was a significant predictor only among women.

## 5. Discussion

As expected, conservatoire musicians reported high intrinsic motivation (H_1_) and low amotivation (H_2_) in instrument practice, which are indispensable conditions for long time commitment, required for reaching higher music education [1, 2]. Furthermore, competence need satisfaction was positively related with intrinsic motivation (H_4_), in line with predictions based on SDT’s cognitive evaluation theory, which posit that IM cannot persist in the absence of perceived competence [17, 70]. Conversely, as anticipated, perceived competence negatively explained amotivation variations in both genders (H_5_), suggesting that the satisfaction of this need may be of central importance to avoid dysregulation tendencies in higher music education.

Conservatoire-level musicians typically initiate their music education at an early age and develop so called careers in serious leisure, strongly rooted in intrinsic motivation based on perceived competence [70–72]. However, in the transition from amateurism into professional education, previous motivation is joined by extrinsic performance goals, externally-mediated rewards, and other-referenced performance standards. The resulting combination of intrinsic and extrinsic motives [23] has mostly been reported as troublesome, showing an opposition rather than additive relationship [4]. In this regard, interestingly, in the present study, higher identified regulation (autonomous EM, or personal goals), predicted higher amotivation levels in both genders. This finding suggests that, notwithstanding its positive status of autonomous motivation [4], under some circumstances, at least for some people, identified regulation may hinder intrinsic motivation in the long run, potentially disrupting the fundaments of long-standing music practice in identity and self.

To put this finding into context, it has been argued that extrinsic gains diminish IM when the latter is assessed as a free-time measure (over-justification hypothesis); but that EM and IM work together to produce higher motivation (additive hypothesis) when IM is assessed as a performance-based measure [31]. Taking this into account, some participants in the present study seemingly experienced a non-additive relationship between their IM and their autonomous EM (identified regulation). Arguably, at first glance, such answers may seem to better represent the typical responses of participants in studies measuring IM as free engagement, rather than as a performance-based measure within professional education. One interpretation for such a finding could be that, even though the questionnaires surveyed “the experience in instrument practice, derived from mandatory lessons”, when responding, participants may have taken into consideration both contextual and situational levels [73, 74]. For instance, when they were asked to consider “why they practised their instrument” (behavioural regulations) and “how competent they felt while practising” (perceived competence), they could have, at least to some extent, taken into account the contextual level (their general liking of musical practice), or even their experience in practising alone at home. Whereas, when they considered “if they felt free to express their opinions” (perceived autonomy), their reference necessarily must have been the situational or state level, involving the specific location, task, teacher and opinion. The hierarchical model of motivation, differentiating situational and contextual levels [73], may thus be useful for future research to determine at what level the opposing relationship between intrinsic and extrinsic motives may be situated.

From a pedagogical perspective, the worst case scenario would be that unfulfilled psychological needs at a situational level (teacher style, design of conservatoire learning activities) may thwart PNS and lead to amotivation at a contextual level, with the unwanted consequence of an adverse effect on career intentions for vocational musicians who had already reached conservatoire levels. Thus, for example, being under the control of a well-regarded teacher may be something that conservatoire students favour. However, if teachers do not explain the rationales behind suggested ways of practice, students will be unable to endorse these behaviours autonomously, and thus, they may experience less autonomy need satisfaction. Whereas, if teachers explain rationales and students endorse these, regulation ceases to be controlled and becomes autonomous.

Furthermore, participants’ scores for introjected regulation (aspect of controlled motivation) were quite high, did not correlate negatively with intrinsic motivation, and were positively related with competence need satisfaction. These results suggest that, in performance-based studies, such as conservatoires, where performance is often judged by other-referenced external standards, controlled motivation may play a role in shaping students’ access to competence need satisfaction, which is indispensable to persevere and counteract amotivation and dropout tendencies [23]: in other words, students first and foremost need to feel competent, and this perception is mediated by the validation from their teachers. This finding may illustrate how conservatoire music teaching tends to gravitate towards a controlling style [75] in hopes of students achieving high performance standards. In this respect, SDT argues that students’ possibilities of experiencing PNS and, thus, (autonomous) quality motivation may be limited by a teaching style strongly centred on extrinsic performance goals and external control [14, 35]. Such controlling teaching may well promote conformity and controlled motivations, like introjected regulation (based on avoiding feelings such as guilt or shame), possibly at the cost of some of the students’ autonomy need satisfaction, autonomous motivation, and self-regulated learning. Students may accept this kind of controlling teaching style, but when they do not understand the rationales behind the types of practices demanded from them, they cannot feel autonomous about these. In this scenario, conservatoire students may experience a trade-off between the need to feel competent by fulfilling their teachers expectations regarding practice behaviours, and the need to feel autonomous by deciding which practice behaviours to enact and how. The only way in which introjected regulation could become truly autonomous (i.e. identified regulation) is through endorsement and understanding of the rationales behind the required actions; in these cases actions that were at first enacted through introjected regulation can become internalized (i.e. identified regulation). This means that even if students have decided to put themselves in the hands of their teachers’ greater experience, they may still feel that practice behaviours demanded by instructors are not fitting their needs and that they cannot influence the ways in which they are supposed to invest their time and effort. If teachers have not explained the rationales, internalization and autonomous re-endorsement of these behaviours may be difficult.

In line with previous findings, however, our results also suggest that, in some cases, controlled motivation, given its link with perceived competence, may (from a quantitative perspective) provide the necessary motivation to avoid dropout [23, 24, 76], even if not quality motivation [4]. As regards the key aspect of difference between introjection and identification, Ryan and Deci [13, p. 188] explain that “regulation through identification is more autonomous or volitional than external or introjected regulation”, given that “in acting out of identified regulations, people are not simply complying with an external or introjected demand but are instead acting out of a belief in the personal importance or perceived value of the activity”, which requires “having understood and personally accepted the value of the acts (whether inhibitions or commission)”. This would mean that, regardless of how typically positive or customary this kind of relationship may be in that domain, identification may demand not just the decision to put oneself under the control of a teacher, but understanding and autonomously internalizing the rationales behind the practice behaviours prescribed by them. As a result, identification facilitates not only a perception of choice but also an internal perceived locus of control (IPLOC), thus, satisfying autonomy need [13].

However, some consensus also exists on the notion that domains in which inter-personal comparisons, competition, achievement, and competence or performance stand out as very important during long periods, external regulations, introjects and ego involvement may be critically relevant [23]. As a matter of fact, recent studies in physical activity have found that girls are more likely than boys to pertain to motivational profiles with high external regulation and that comparing among the two profiles with high autonomous motivation (intrinsic and identified), the one ruled additionally by very high introjection exceeds the purely autonomy-driven profile in their competence levels, “showing the advantages of a profile that includes high levels of introjection for activities including at least some aspects that that are not necessarily pleasant for everyone” [77, p. 622].

For behaviours that are initially externally regulated by controlled motivations to become autonomous, internalization processes need to be supported adequately [16] through learning environments providing PNS. For instance, competence need may be supported when teachers praise efforts and strategies, rather than outcomes and abilities; and it can be thwarted by perfectionistic standards, social comparisons and norm-based evaluation criteria [4]. Similarly, autonomy need can be supported by providing rationales for instructions, acknowledging students’ feelings, giving them choice of repertoire, and assisting them in setting their own goals in practice; whereas it can be thwarted by focusing strictly on performance, teacher control, rules, and rewards or punishments [4].

Regarding gender-differences in maladjustment in conservatoire instrument practice, as expected, amotivation was more pronounced among women (H_3_). Furthermore, our results showed that perceived competence was marginally lower among women conservatoire students than among men. This may in part be due to women musicians’ autonomous high self-demand levels [53], which in turn, may lead them to perceived task difficulty as high [51]. Furthermore, some women musicians may feel a greater gap between their self-perceived skills and their ideal skill levels, thus, potentially experiencing lower self-efficacy [44] and less positive attitudes towards their own skills [55].

Also, both the proportion of amotivation variations explained by study variables, and the negative beta weights of psychological needs satisfaction on these variations, were more robust among women musicians. Furthermore, autonomy need satisfaction, which has been observed to be distinctly relevant among women students [34], was a significant negative predictor of amotivation variations (H_6_) only among women. In this regard, our findings suggest that psychological needs that are left unfulfilled by learning environments (especially autonomy need) may explain maladaptive dysregulation tendencies to a greater extent among women musicians [53]. These results are also reasonable, since men typically have been observed to take a more flexible, adaptive and pragmatic approach to practice [55], possibly experiencing less loss of perceived autonomy than women.

Alternative explanations, which would need to be considered by future studies, would be that higher levels of anxiety regarding academic evaluations have been found in women who are more prone to maladaptive perfectionism and avoidance goal orientations [78]. Furthermore, also personality traits could be taken into account when analysing person-context interactions. For example, women have been observed to score higher than men on neuroticism and agreeableness, and also on some aspects of conscientiousness, such as order, dutifulness, and self-discipline [79]. With these trait-level differences in mind, the present findings could mean that women students’ high tendency towards autonomous, strict and rigorous self-regulated learning style [55], added to their tendency to conform to teachers’ norms and standards, may make them experience controlling teaching style [34] as a severe challenge to their perceived autonomy [55] to a greater extent than men. This may explain more frequent negative experiences like higher stress appraisals and disengagement coping strategies, potentially influencing their career intentions adversely [35], likely in close relation with a thwarted psychological need for autonomy. The fact that controlling teaching style may compromise women’s autonomy need satisfaction to a greater extent than men’s, causing women to experience more stress and anxiety, may derive from the disconnection between teacher demands and the ways in which women musicians had planned to practice beforehand. This disconnection could be less dramatic in the case of men musicians, given that these have not planned or decided in advance how they want to practice, protecting them from a perceived loss of autonomy.

## 6. Conclusions and limitations of the study

Several conclusions can be drawn from our findings. Firstly, context-derived satisfaction of the need for competence is vital for developing and maintaining intrinsic and quality motivation, and for avoiding amotivation in advanced music practice. Secondly, however, the coexistence of intrinsic motives with autonomous yet extrinsic motives, such as identified regulation (personal goals), may be experienced as troublesome by some conservatoire students, potentially leading to amotivation in the transition into professional education (in both genders). Thirdly, notwithstanding the close link between IM and competence need satisfaction, perceived competence may also be positively related with controlled extrinsic regulations, such as introjected regulation, given that these are closely linked with access to competence need satisfaction, through external validation of achievement of performance standards.

Lastly, to our knowledge, the present study is one of the first to assess gender-differences in amotivation in conservatoire instrument practice, linking these with musicians’ psychological need satisfaction. In this regard, women musicians may be perceiving that PNS, especially of the need for autonomy, is more closely linked with amotivation than their men counterparts. This suggests that person-context interactions, between women’s higher autonomous learning style, and conservatoire’s externally controlling teaching style, could to some extent account for gender-differences in the regression models predicting amotivation variations.

The present study had to deal with several limitations. The relatively small sample size (*N* = 141) was not ideal, though sufficient, as observed in the statistical analyses. Also, related to the relatively small sample size, the present contribution could not rigorously address the influence of instrument played on amotivation, as dividing participants into study courses, or instruments played would be problematic. Nonetheless, we provide a general idea of participants who are practitioners of specific instruments, or music styles. Lastly, the present contribution could not take into consideration if the teachers were male or female, given that registering the gender of these teachers would have identified both teachers and students impeding anonymity. Future studies should consider controlling for the effects of sex within teacher-student dyads.

Despite these limitations, our findings have some important implications for theory and practice. On the theoretical level, even though psychological needs are universal, thus, ubiquitous and independent of individuals opinions about their relative importance [21], the ways in which these needs are satisfied or thwarted depend on the person. For example, faced with uncertainty, scientists satisfy their competence and autonomy needs by autonomously searching for valid and reliable information to form their–more or less technical–judgements; whereas people who lack a scientific vain or even deny science also seek to satisfy these same needs by autonomously embracing situational judgements–aligned with their prevalent beliefs–in order to feel competent in maintaining a sense of control. It is self-evident that these two contrasting ways of coping with uncertainty depend on peoples’ beliefs and value systems, on which in turn their attitudes and behaviours rest. Similarly, it has been shown that women and men conservatoire music students seemingly diverge in their practice behaviours and attitudes towards their own learning styles and skills [44, 47, 55], which implies that–without an alignment of these beliefs, values, and conducts–satisfaction of universal psychological needs cannot occur in the same ways for both genders.

On a practical level, in order to facilitate needs satisfaction for women and men conservatoire students alike, music teachers and coaches seeking to be inclusive may need to assess their students’ academic beliefs and values, as well as their learning styles, in order to design more tailored strategies for facilitating their needs satisfaction, or–alternatively–promoting specific rationales for the prescribed ways of practising, that may exert effects on students’ academic beliefs and value formation, which ultimately may get students aligned with canonical or suggested ways of performing and learning.
